# The Combination of High Levels of Adiponectin and Insulin Resistance Are Affected by Aging in Non-Obese Old Peoples

**DOI:** 10.3389/fendo.2021.805244

**Published:** 2022-01-07

**Authors:** Jun Muratsu, Kei Kamide, Takashi Fujimoto, Yasushi Takeya, Ken Sugimoto, Yoshiaki Taniyama, Atsuyuki Morishima, Katsuhiko Sakaguchi, Yuji Matsuzawa, Hiromi Rakugi

**Affiliations:** ^1^ Department of Nephrology and Hypertension, Sumitomo Hospital, Osaka, Japan; ^2^ Department of Nephrology, Rinku General Medical Center, Izumisano City, Japan; ^3^ Division of Health Sciences, Osaka University Graduate School of Medicine, Osaka, Japan; ^4^ Department of Geriatric and General Medicine, Osaka University Graduate School of Medicine, Suita, Japan; ^5^ Department of General and Geriatric Medicine, Kawasaki Medical School General Medical Center, Okayama, Japan

**Keywords:** adiponectin, insulin resistance, aging, body mass index, old peoples

## Abstract

**Background:**

Adipokine dysregulation is a key feature of insulin resistance and a metabolic syndrome associated with obesity. Low adiponectin levels are associated with higher risks of cardiovascular diseases (CVD). However, high adiponectin levels have also been associated with increased all-cause and cardiovascular mortality in the elderly. This adiponectin paradox has yet to be clarified, which has hindered our understanding of the biological role of adiponectin. Adipokine dysregulation and insulin resistance are also associated with energy-deprivation conditions, such as frailty in old age. The objective of this study was to investigate the association between plasma adiponectin and insulin resistance using the homeostasis model assessment for insulin resistance (HOMA-IR) classified by age. In particular, we sought to determine the factors of the subjects associated with both high adiponectin levels and HOMA-IR (H-adiponectin/H-HOMA) and high adiponectin levels and low HOMA-IR (H-adiponectin/L-HOMA).

**Methods:**

The eligible subjects in this cross-sectional study were 33,216 individuals who had undergone health checkups at the Physical Checkup Center of Sumitomo Hospital between April 2008 and December 2018. After excluding 26,371 individuals who were under 60 years old, 529 who had been taking medications for diabetes mellitus, and 690 with missing data, the present study included 5,673 (3,467 males, 2,206 females) subjects with no missing data. The relationship between serum adiponectin levels and HOMA-IR was assessed using logistic regression models adjusted by clinically relevant factors.

**Results:**

In the multivariable logistic regression analysis, age and low BMI were shown to positively correlate with the characteristics of H-adiponectin/H-HOMA. In females, systolic blood pressure was also shown to be an associated factor.

**Conclusion:**

In conclusion, this study showed that aging or a low BMI may contribute to high adiponectin levels and insulin resistance.

## Introduction

Adiponectin, an adipocyte-derived cytokine, reduces levels of blood free fatty acids (FFAs) and has been associated with improved lipid profiles, better glycemic control, and reduced inflammation in diabetic patients (1). Conversely, adiponectin has also been associated with increased risks of diabetes and hypertension (2–5). A number of observations suggest that adiponectin deficiency plays a role in the development of insulin resistance and subsequent type 2 diabetes (6), with lower adiponectin levels more closely related to the degree of insulin resistance and hyperinsulinemia than to the degree of adiposity and glucose intolerance (7). Therefore, in general, a high adiponectin level is associated with a favorable cardiovascular disease (CVD) risk profile (8–10). However, the relationship between adiponectin levels and CVD is not consistent, with some studies reporting that high adiponectin levels are associated with increased all-cause and CVD mortality (11, 12). It has been hypothesized that this discrepancy may be related to the aging process in humans, and previous reports have noted that aging may be associated with elevated serum adiponectin levels in healthy adults, despite the higher CVD risk in elderly individuals (13–17). Furthermore, there is the possibility that high serum adiponectin may be associated with frailty in the elderly (18). Since frailty and sarcopenia can lead to insulin resistance in these individuals, insulin resistance could be a key player in the complex relationship among serum adiponectin levels, aging, and CVD, which is referred to as “the adiponectin paradox” (19). The aim of this study was to clarify the underlying mechanisms in the adiponectin paradox using health checkup data from a large sample size, which included assessment of serum adiponectin levels by age stratification and with a focus on insulin resistance.

## Methods

### Study Design

**Figure 1:**
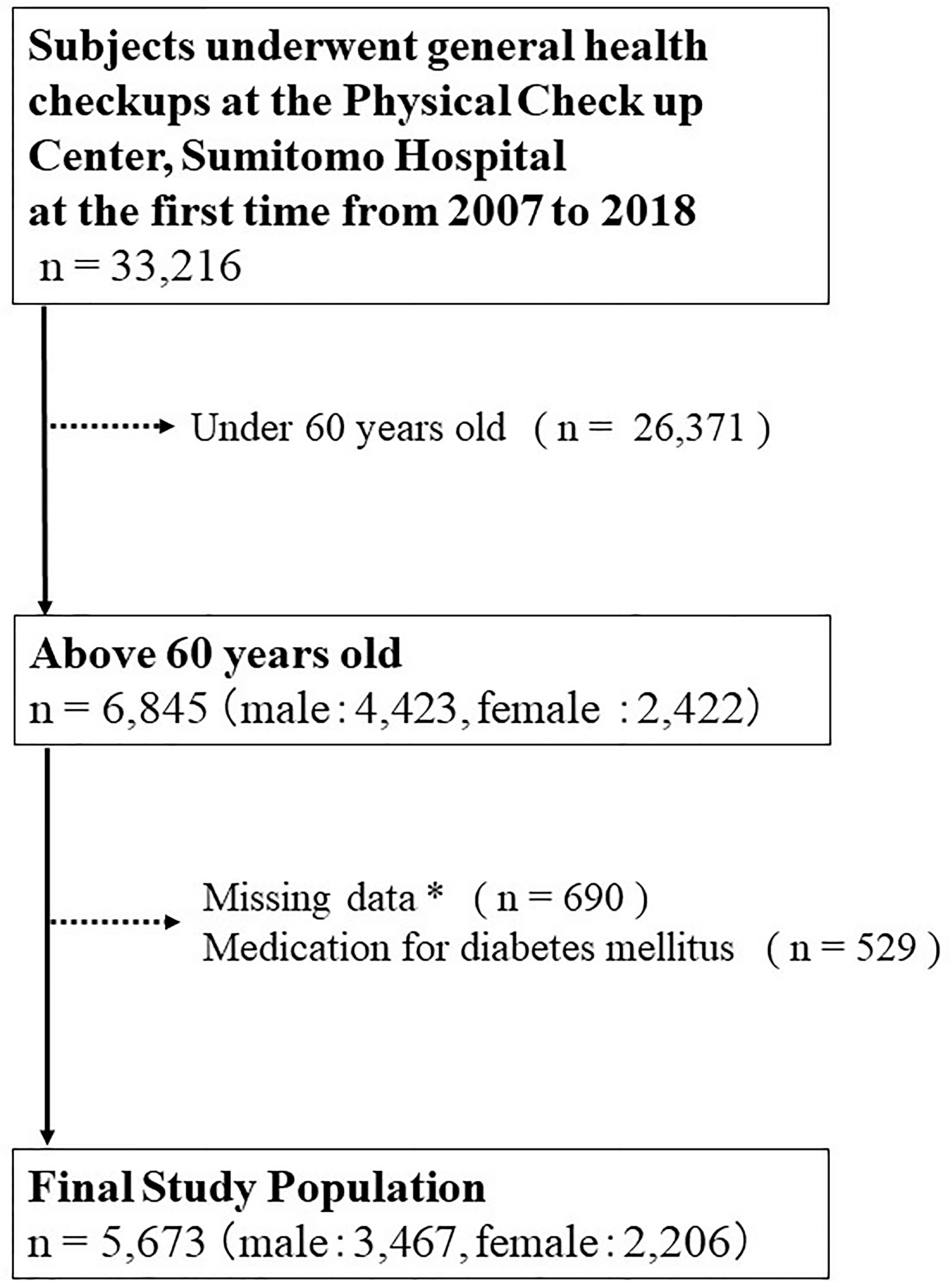
Inclusion and exclusion processes of the present study. * Including age, sex, body mass index, waist circumference, systolic blood pressure, diastolic blood pressure, hemoglobin, aspartate transaminase, alanine aminotransferase, albumin, total cholesterol, triglyceride, high-density lipoprotein cholesterol, low-density lipoprotein cholesterol, fasting blood sugar level, creatinine, uric acid, estimated glomerular filtration rate (e-GFR), hemoglobin A1c, insulin, adiponectin, smoking habits, alcohol consumption per day, medical history of hypertension, diabetes mellitus, dyslipidemia, stroke, hyperuricemia or coronary artery disease.

The eligible subjects in this cross-sectional study were 33,216 individuals who had undergone health checkups at the Physical Checkup Center of Sumitomo Hospital between April 2008 and December 2018 (**Figure 1**). The health checkup program is designed to detect diseases at early stages. After excluding 26,371 individuals under 60 years old, 529 who had been taking medications for diabetes mellitus, and 690 with missing data, the present study included 5,673 (3,467 males, 2,206 females) subjects with no missing data. We excluded individuals who had been taking medications for diabetes mellitus because it had been previously reported that the antidiabetic drug pioglitazone is associated with high adiponectin levels (20).

The study was approved by the human ethics committees of Sumitomo Hospital and was conducted according to the principles of the Declaration of Helsinki (approval No. 2021-38). Written informed consent to provide medical information and blood samples was obtained before the checkup examinations from all participants, and each subject had the right to refuse the use of their results.

### Clinical and Laboratory Assessment

The subjects’ height and weight were measured while they were wearing light underwear and without shoes, and their waist circumferences were measured at the level of the umbilicus. Blood pressures were measured using an automatic sphygmomanometer (BP-203RVIII; Colin, Tokyo, Japan) while the subjects were sitting. Information on their medical histories was evaluated using standardized self-administered questionnaires and interviews by doctors. Laboratory data and adiponectin levels were measured after overnight fasting. In Japan, the measurement method for HbA1c has changed from JDS to NGSP. Therefore, all HbA1c (JDS) data were converted from JDS to NGSP according to the guidelines of the Japan Diabetes Society as follows: HbA1c (NGSP) (%) = 1.02 × HbA1c (JDS) (%) + 0.25% (21). To calculate eGFR, the Japanese formula was used (eGFR [mL/min/1.73 m2] = 194 × serum creatinine [mg/dL] −1.094 × age [year] − 0.287 × 0.739 [for females]) (22). Serum adiponectin levels, which included both low-molecular weight (LMW) isoforms (approximately 30-70 kDa) and high-molecular weight (HMW) isoforms (12-, 18-mers), were measured by a latex particle-enhanced turbidimetric immunoassay (human adiponectin latex kit; Otsuka Pharmaceutical Co. Ltd, Tokyo, Japan) as previously reported (23). Adiponectin is inversely correlated with visceral adiposity (13), and it has been demonstrated that hypoadiponectinaemia is associated with insulin resistance (24). On the other hand, the homeostasis model assessment for insulin resistance (HOMA-IR) is often used in clinical practice as an index of insulin resistance and is 104 calculated as follows: HOMA-IR = fasting blood sugar (mg/dL) × IRI/405 (25, 26). HOMA-IR reflects both the presence and extent of any insulin resistance and is a terrific way of revealing the dynamic between fasting blood sugar and responsive insulin. Theoretically, if the level of adiponectin is high, HOMA-IR will be low. However, it is possible for subjects to have both high adiponectin and HOMA-IR, and our aim was to determine the factors that give rise to this.

### Statistical Analysis

Comparisons between two groups were analyzed using an unpaired *t*-test or the Mann-Whitney *U* test for continuous variables and the *χ^2^* test for categorical variables. The level of serum adiponectin is affected by sex, body fat mass, several pathological factors or therapeutic interventions, and possibly age (13). Since the values of adiponectin levels differ depending on sex, the median values were calculated and divided into four groups: high adiponectin + high HOMA-IR (H-adiponectin/H-HOMA); high adiponectin + low HOMA-IR (H-adiponectinL-HOMA); low adiponectin + high HOMA-IR (L-adiponectin/H-HOMA); and low adiponectin + low HOMA-IR (L-adiponectin/L- HOMA). These groups were used to assess the potential role of adiponectin in aging. The median levels of adiponectin were 7.9 and 13.2 μg/mL for males and female, respectively, while median HOMA-IR values for males and females were 1.32 and 1.18. We defined adiponectin levels above 7.9 μg/mL in males and 13.2 μg/mL in females as high (H-adiponectin). Conversely, adiponectin levels less than 7.9 μg/mL in males and 13.2 μg/mL in females were defined as low (L-adiponectin). In addition, we defined HOMA-IR levels above 1.32 in males and 1.18 in females as high (H-HOMA) and levels less than 1.32 in males and 1.18 in females as low (L-HOMA). To assess the characteristics of the subjects who were H-adiponectin/H-HOMA, their odds ratios were calculated in an adjusted multivariable logistic regression model and divided by sex. In addition, the association of age with H-adiponectin/H-HOMA and L-adiponectin/H-HOMA was assessed in three subgroups with BMI values of <22.0, 22.0-24.9, and ≥25 kg/m2, and by sex. Each odds ratio value indicated a degree of influence on H-adiponectin/H-HOMA. P-values < 0.05 were considered to be statistically significant. Categorical variables are expressed as numbers (percentages), and continuous variables are shown as means ± standard deviation or medians (interquartile range), as appropriate. All statistical analyses were performed using Stata version 14.2 (Stata Corp, http://www.stata.com).

## Results

**Figure 2:**
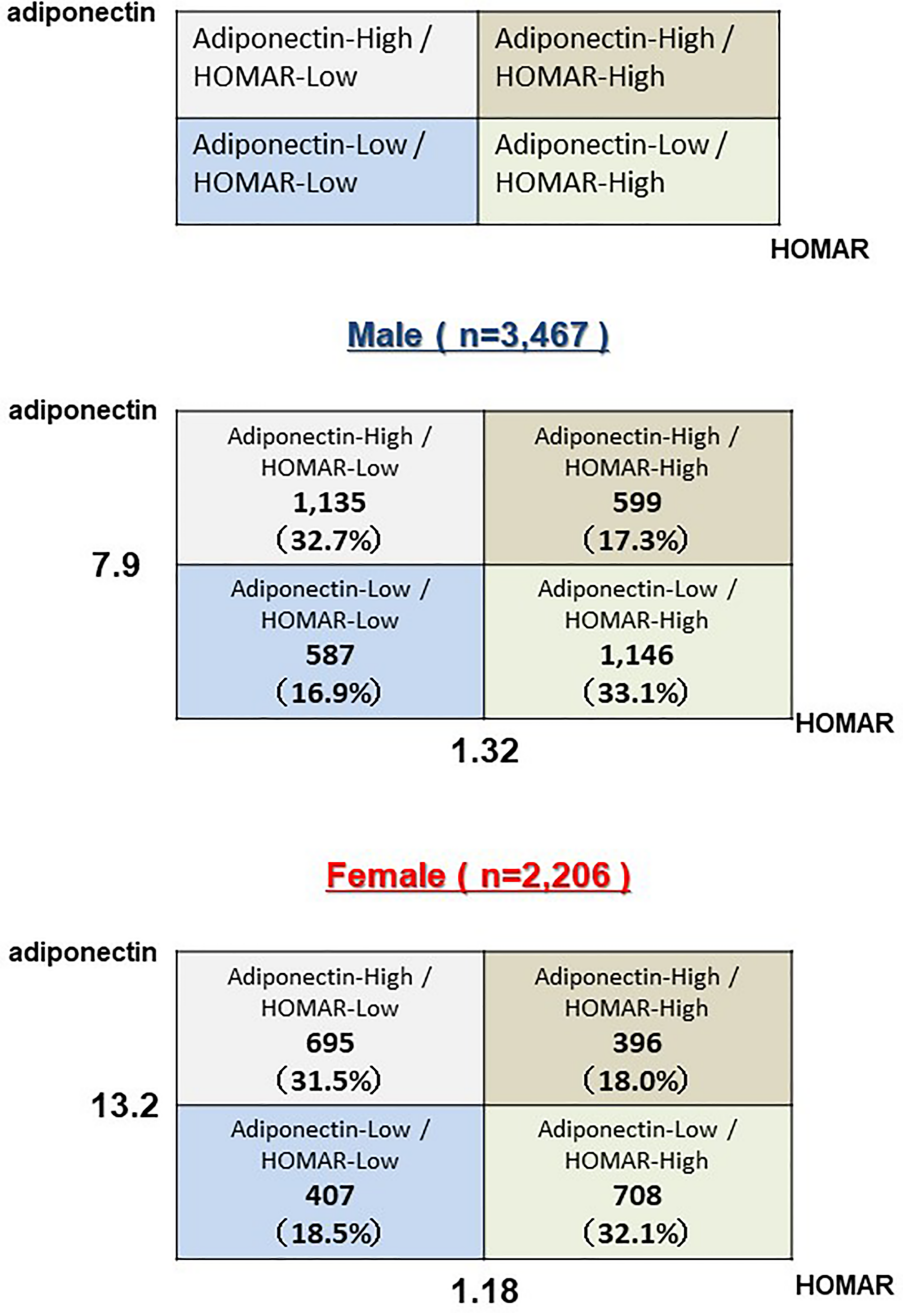
Subjects above 60 years old were divided into four groups (H-adiponectin/H-HOMA, H-adiponectin/L-HOMA, L-adiponectin/H-HOMA, L-adiponectin/L-HOMA) by median adiponectin and HOMA-IR values and by sex.

Baseline clinical characteristics of this study are shown in **Table 1**. The 5,673 subjects included 3,467 males and 2,206 females, with average ages of 66 and 67, respectively. Medical histories of hypertension, hyperuricemia, or coronary artery disease were shown to be significantly higher in males than females. On the other hand, a medical history of dyslipidemia was shown to be significantly higher in females. The females also showed a higher prevalence of anemia and lower BMI values, while males had lower levels of total cholesterol. The median levels of adiponectin were 7.9 and 13.2 μg/mL for males and females, respectively, while median HOMA-IR values for males and females were 1.32 and 1.18. Based on these results, we divided all subjects into the H-adiponectin/H-HOMA, H-adiponectin/L-HOMA, L-adiponectin/H-HOMA, and L-adiponectin/L-HOMA groups. For males, these groups comprised 599 (17.3%), 1,135 (32.7%), 1,146 (33.1%), and 587 (16.9%) subjects, respectively, while the corresponding groups for females comprised 396 (18.0%), 695 (31.5%), 708 (32.1%), and 407 (18.5%) subjects (**Figure 2**). In order to clarify the characteristics associated with H-adiponectin/H-HOMA, we compared the subjects in the H-adiponectin/H-HOMA and H-adiponectin/L-HOMA groups (**Tables 2A**, **2B**) and found that the H-adiponectin/H-HOMA subjects were older. However, body weight, BMI, waist circumference, and hemoglobin levels were significantly lower in the H-adiponectin/H-HOMA group. With regard to the subjects’ medical histories, no significant differences were seen between the subjects in the two groups.

**Table 1:** Clinical characteristics of 5,673 subjects above 60 years old stratified by sex.

Parameters Total n= 5,673	male n = 3,467	female n = 2,206	p value
Age, years	66 ± 6	67 ± 6	0.006
Height, cm	167.5 ± 5.8	154.2 ± 5.5	<0.001
Weight, kg	65.8 ± 9.0	51.7 ± 8.1	<0.001
Body mass Index, kg/m^2^	23.4 ± 2.8	21.8 ± 3.2	<0.001
**Medical History**			
Hypertension	1,194 (34.4)	536 (24.3)	<0.001
Stroke	109 (3.1)	56 (2.5)	0.186
Coronary artery disease	180 (5.2)	70 (3.2)	<0.001
**Physical findings on admission**			
Systolic blood pressure, mmHg	128 ± 16	126 ± 17	<0.001
Diastolic blood pressure, mmHg	80 ± 10	77 ± 11	<0.001
**Laboratory Data on admission**			
Hemoglobin, mg/dL	14.7 ± 1.1	13.3 ± 0.9	<0.001
Albumin, mg/dL	4.4 ± 0.3	4.4 ± 0.3	0.250
Total chlesterol, mg/dL	213 ± 32	232 ± 34	<0.001
Triglyceride, mg/dL	131 ± 88	104 ± 64	<0.001
High-density lipoprotein cholesterol, mg/dL	61 ± 15	73 ± 17	<0.001
Low-density lipoprotein cholesterol, mg/dL	127 ± 30	136 ± 31	<0.001
Creatinine, mg/dL	0.88 ± 0.23	0.65 ± 0.12	<0.001
Uric acid, mg/dL	6.2 ± 1.2	4.8 ± 1.1	<0.001
eGFR, mL/min/1.73m^2^	69.8 ± 13.2	71.9 ± 13.5	<0.001
Hemoglobin A1c (NGSP), %	5.8 ± 0.6	5.7 ± 0.5	0.024
Insulin, μIU/mL	6.33 ± 4.23	5.97 ± 4.00	<0.001
HOMA-R	1.32 (0.88, 2.02)	1.18(0.80,1.78)	<0.001
Adiponectin, μg/mL	7.9 (5.7, 10.9)	13.2 (9.5, 18.6)	<0.001

Categorical variables are expressed as numbers (percentages) and continuous variables are shown as mean ± standard deviation or median (interquartile range), as appropriate. eGFR, estimated glomerular filtration rate.

**Table 2A:** Clinical and biological characteristics of high HOMA-R 1,745 males stratified by H-adiponectin/H-HOMA and H-adiponectin/L-HOMA.

Parameters	High adiponectin High HOMA-R n = 599	Low adiponectin High HOMA-R n = 1,146	p value
Age, years	67 ± 6	65 ± 5	<0.001
Height, cm	167.4 ± 6.1	167.9 ± 5.6	0.123
Weight, kg	67.6 ± 8.8	70.3 ± 8.2	<0.001
Body mass Index, kg/m^2^	24.1 ± 2.6	24.9 ± 2.6	<0.001
**Medical History**			
Hypertension	268 (44.7)	487 (42.5)	0.369
Stroke	16 (2.7)	39 (3.4)	0.406
Coronary artery disease	36 (6.0)	70 (6.1)	0.935
**Physical findings on admission**			
Systolic blood pressure, mmHg	130 ± 16	131 ± 15	0.407
Diastolic blood pressure, mmHg	81 ± 10	81 ± 10	0.220
**Laboratory Data on admission**			
Hemoglobin, mg/dL	14.7 ± 1.1	15.0 ± 1.1	<0.001
Albumin, mg/dL	4.4 ± 0.3	4.5 ± 0.3	<0.001
Total chlesterol, mg/dL	212 ± 34	213 ± 33	0.773
Triglyceride	125 ± 62	163 ± 100	<0.001
High-density lipoprotein cholesterol	61 ± 14	54 ± 12	<0.001
Low-density lipoprotein cholesterol	128 ± 31	132 ± 30	0.128
Creatinine, mg/dL	0.92 ± 0.37	0.88 ± 0.20	0.007
Uric acid, mg/dL	6.1 ± 1.2	6.4 ± 1.2	<0.001
eGFR, mL/min/1.73m^2^	67.3 ± 13.6	69.9 ± 13.0	<0.001
Hemoglobin A1c (NGSP), %	5.8 ± 0.6	5.9 ± 0.8	0.016
Insulin, μIU/mL	8.01 ± 3.51	9.50 ± 4.86	<0.001
HOMA-R	1.80 (1.51, 2.28)	2.12 (1.66, 2.96)	<0.001
Adiponectin, μg/mL	10.1 (8.9, 12.4)	5.6 (4.6, 6.6)	<0.001

Categorical variables are expressed as numbers (percentages) and continuous variables are shown as mean ± standard deviation or median (interquartile range), as appropriate. eGFR, estimated glomerular filtration rate.

**Table 2B:** Clinical and biological characteristics of high HOMA-R 1,104 females stratified by H-adiponectin/H-HOMA and H-adiponectin/L-HOMA.

Parameters	High adiponectin High HOMA-R n = 396	Low adiponectin High HOMA-R n = 708	p value
Age, years	69 ± 7	67 ± 6	<0.001
Height, cm	153.2 ± 5.5	154.1 ± 5.5	0.035
Weight, kg	52.2 ± 7.8	56.2 ± 8.5	<0.001
Body mass Index, kg/m^2^	22.2 ± 3.1	23.6 ± 3.2	<0.001
**Medical History**			
Hypertension	117 (29.5)	242 (34.2)	0.115
Stroke	10 (2.5)	22 (3.1)	0.580
Coronary artery disease	9 (2.3)	31 (4.4)	0.073
**Physical findings on admission**			
Systolic blood pressure, mmHg	131 ± 17	131 ± 17	0.559
Diastolic blood pressure, mmHg	79 ± 10	79 ± 10	0.644
**Laboratory Data on admission**			
Hemoglobin, mg/dL	13.3 ± 1.0	13.5 ± 0.9	<0.001
Albumin, mg/dL	4.4 ± 0.3	4.4 ± 0.3	0.004
Total chlesterol, mg/dL	234 ± 35	228 ± 35	0.003
Triglyceride	98 ± 46	132 ± 88	<0.001
High-density lipoprotein cholesterol	76 ± 16	64 ± 15	<0.001
Low-density lipoprotein cholesterol	135 ± 32	139 ± 32	0.012
Creatinine, mg/dL	0.66 ± 0.14	0.65 ± 0.11	0.421
Uric acid, mg/dL	4.8 ± 1.1	5.2 ± 1.0	<0.001
eGFR (mL/min/1.73m^2^)	70.3 ± 14.5	71.7 ± 13.6	0.161
Hemoglobin A1c (NGSP), %	5.7 ± 0.4	5.9 ± 0.6	<0.001
Insulin, μIU/mL	7.87 ± 3.75	8.76 ± 4.57	<0.001
HOMA-R	1.63 (1.37, 2.17)	1.87 (1.49, 2.49)	<0.001
Adiponectin, μg/mL	17.55 (15, 22)	9.1 (7.1, 11)	<0.001

Categorical variables are expressed as numbers (percentages) and continuous variables are shown as mean ± standard deviation or median (interquartile range), as appropriate. eGFR, estimated glomerular filtration rate.

To investigate the factors associated with H-adiponectin/H-HOMA, we obtained odds ratios with an adjusted multivariable logistic regression model (**Table 3**) and found age and BMI to be associated factors. In females, systolic blood pressure also showed a positive correlation. Renal function affects adiponectin levels (27). However, decreased levels of eGFR were shown in many old peoples.

**Table 3:** Logistic multivariable regression analysis for the combination of high adiponectin and high HOMA-R.

	Male		Female	
	Odds ratio (95% CI)	p-value	Odds ratio (95% CI)	p-value
Age	1.04 (1.03-1.06)	<0.001	1.07 (1.05-1.09)	<0.001
Body mass Index	1.12 (1.08-1.16)	<0.001	1.07 (1.03-1.11)	0.001
Systolic blood pressure	1.00 (0.99-1.01)	0.103	1.16 (1.01-1.02)	<0.001
HbA1c(NGSP)	1.06 (0.92-1.23)	0.432	0.66 (0.50-0.87)	0.004
Triglyceride	0.99 (0.99-0.99)	0.019	1.00 (1.00-1.00)	0.126
High-density lipoprotein cholesterol	1.00 (0.99-1.01)	0.168	1.02 (1.01-1.02)	<0.001
Hemoglobin	1.05 (0.96-1.15)	0.270	1.09 (0.96-1.24)	0.182
albumin	0.99 (0.69-1.42)	0.963	0.92 (0.58-1.46)	0.731
eGFR	0.99 (0.98-0.99)	<0.001	1.00 (0.99-1.00)	0.087
**Medical history**				
Coronary artery disease	1.02 (0.69-1.51)	0.917	0.53 (0.26-1.10)	0.087
Stroke	0.68 (0.39-1.17)	0.163	0.81 (0.39-1.65)	0.559

Adjusted for age (y), Body mass index (kg/m^2^), Systolic blood pressure (mmHg), HbA1c (%), Triglyceride, High-density lipoprotein cholesterol, Hemoglobin, Albumin, estimated glomerular filtration rate (eGFR) (mL/min/1.73 m^2^), and medical history of coronary disease and stroke at their first visit during the study period.

Therefore, in logistic multivariable regression model, eGFR were adjusted as confounding factor. In addition, we performed additional analysis without renal disease and/or eGFR < 60 mL/min/1.73m2 cases. Age and BMI showed a positive correlation as the previous analysis included kidney disease in **Supplement Table A**.

To clarify the effect of BMI on the association between age and H-adiponectin/H-HOMA, logistic regression analysis was performed after dividing the subjects based on BMI levels. Interestingly, age was positively correlate in BMI <25 kg/m2 (**Table 4**). On the other hand, to clarify the effect of age on the association between BMI and H-adiponectin/H-HOMA, logistic regression analysis was performed after dividing the subjects based on age. Both men and women with age < 70 years old showed a positive correlation with BMI, although men with age ≥ 75 years old also showed a positive correlation (**Supplement Table B**).

**Table 4:** Logistic multivariable regression analysis for High adiponectin and High HOMA-R in 5,673 subjects (3,467 males and 2,206 females) stratified by body mass index.

	BMI <22.0				22.0 ≤BMI <25.0				25.0 ≤ BMI			
	Univariable		*Multivariable		Univariable		*Multivariable		Univariable		*Multivariable	
	Odds ratio (95% CI)	*P* value	Odds ratio (95% CI)	*P* value	Odds ratio (95% CI)	*P* value	Odds ratio (95% CI)	*P* value	Odds ratio (95% CI)	*P* value	Odds ratio (95% CI)	*P* value
**Males**												
age	1.04 (1.01-1.07)	0.010	1.04 (1.01-1.08)	0.012	1.06 (1.03-1.08)	<0.001	1.05 (1.03-1.08)	<0.001	1.04 (1.01-1.07)	0.004	1.03 (0.99-1.06)	0.068
BMI	1.42 (1.20-1.69)	<0.001	1.35 (1.13-1.61)	0.001	1.07 (0.92-1.25)	0.358	1.13 (0.97-1.33)	0.124	0.99 (0.90-1.08)	0.758	1.00 (0.92-1.10)	0.918
**Females**												
age	1.08 (1.05-1.10)	<0.001	1.08 (1.05-1.10)	<0.001	1.06 (1.03-1.10)	<0.001	1.06 (1.03-1.10)	0.001	1.04 (1.00-1.08)	0.032	1.04 (0.99-1.08)	0.103
BMI	1.07 (0.97-1.18)	0.154	1.08 (0.97-1.19)	0.154	0.97 (0.77-1.23)	0.806	0.93 (0.73-1.19)	0.579	0.84 (0.73-0.97)	0.015	0.85 (0.73-0.99)	0.049

CI, confidence intercal.

*Adjusted for age (y), Body mass index (kg/m^2^), Systolic blood pressure (mmHg), HbA1c (%), Triglyceride, High-density lipoprotein cholesterol, Hemoglobin, Albumin, estimated glomerular filtration rate (eGFR) (mL/min/1.73 m^2^), and medical history of coronary disease and stroke at their first visit during the study period.

## Discussion

Previous studies have reported that age is associated with elevated serum adiponectin levels in healthy adults, despite the higher cardiovascular risk in elderly individuals (13–17, 28). In animal studies, mice with fat-specific disruption of the insulin receptor gene (FIRKO mice) are reported to have decreased adiposity, lower fasting insulin levels, and an extended lifespan. In addition, FIRKO mice also have elevated serum adiponectin levels (29, 30). Several reports have indicated the occurrence of hyper-adiponectinemia in centenarians and an inverse correlation between adiponectin and HOMA-IR (31).

However, the mechanisms underlying the age-related increase in serum adiponectin is unclear. While several reports have examined the relationship between adiponectin and age in association with visceral fat (13, 15), renal function (14, 16, 32), or sex hormones (14, 28), it remains unclear whether HOMA-IR is associated with serum adiponectin levels in the elderly. On the basis of these findings, age-related changes in serum adiponectin should be taken into account when metabolic changes are evaluated using serum adiponectin levels in clinical practice.

This study revealed that H-adiponectin/H-HOMA may be significantly associated with aging. In addition, our results showed that subjects with H-adiponectin/H-HOMA may be undernourished when compared with H-aiponectin/L-HOMA subjects. Age was shown to positively correlate with BMI values <25 kg/m2 but not with values ≥25 kg/m2 (**Table 4**). It has been reported that a low BMI can reflect reduced muscle mass (33, 34). On the other hand, both men and women with age < 70 years old showed a positive correlation with BMI, although men with age ≥ 75 years old also showed a positive correlation. This indicated obesity was mainly associated with the combination of high levels of adiponectin and insulin resistance below 70 years old. However, above 70 years old, other factors may strongly be associated with the combination of high levels of adiponectin and insulin resistance. Taken together, this suggest that adiponectin-independent pathways may also be involved in insulin resistance in the elderly.

A previous report examined 44 subjects using the euglycemic hyperinsulinemic glucose clamp test and observed atherosclerotic change by ultrasonography of the heart and carotid arteries. There was a significant negative correlation between aging and insulin sensitivity, while a significant increase in left ventricular mass index and carotid wall thickening accompanied by insulin resistance were shown in non-elderly subjects but not in elderly subjects. From these results, it was concluded that aging decreases insulin sensitivity even in essential hypertensive subjects and that insulin resistance does not affect the progression of cardiac hypertrophy or atherosclerosis in elderly subjects with essential hypertension (18). This, however, does not rule out the possibility that atherosclerosis is involved in insulin resistance in elderly people. In our logistic multivariable study, neither atherosclerotic disease, stroke, nor coronary artery disease were significantly associated with H-adiponectin/H-HOMA. Instead, we found that aging, undernutrition, and sarcopenia may contribute to H-adiponectin/H- HOMA. Indeed, a recent study with subjects aged approximately 83 years indicated that higher plasma adiponectin levels are associated with frailty status in older Japanese adults in the general population (35). Furthermore, a previous report found that centenarians have increased adiponectin levels and that adiponectin levels are inversely correlated with BMI, waist circumference, and the percentage of body fat. The researchers also found that two common variants of adiponectin gene *ADIPOQ* are associated with higher adiponectin levels and longevity (36). It has also been reported that serum adiponectin may protect against sarcopenia (37) and that adiponectin up-regulates the phosphatidylinositol 30-kinase-AKT pathway, which promotes muscle protein synthesis and prevents muscle protein degradation (38). Adipokine dysregulation is associated with wasting syndromes such as cachexia and sarcopenia, suggesting that adipose endocrine function is essential for maintaining whole-body energy homeostasis, which is indispensable for a multitude of physiological functions under both energy excess and deprivation conditions (39). Since our study subjects were aged 60 years and older, aging may affect insulin resistance from around 60 years old.

In the previous cohort study, the association of adiponectin with the factors studied was strikingly similar for men and women. Sex differences in circulating adiponectin levels in older adults cannot be explained by sex hormone regulation (40). Our study showed that systolic blood pressure was significantly associated with H-adiponectin/H-HOMA in women. Some reports indicated that high adiponectin levels failed to protect against the development of hypertension in menopausal women. Involvement of adiponectin in autoimmune complex with loss of antioxidative-antiatherogenic properties may be underlying (41). In addition, the other reports showed that in late postmenopausal women with normal renal function, high adiponectin level is associated with favorable lipid profiles (42). Our study showed that HDL-C was positively associated with H-adiponectin/H-HOMA in women. There was possibility that HDL-C may be compensatory or protective for H-adiponectin/H-HOMA in postmenopausal women. Previous reports indicated that trunk fat and leg fat were oppositely associated with adiponectin in older men and women (43). Further studies are needed about fat distribution associated with H-adiponectin/H-HOMA.

The present study had several limitations. First, lifestyle behaviors and medical histories were evaluated using a self-administered questionnaire, and participants may have overstated the healthiness of their lifestyles (44). Further evaluation of these factors based on an established questionnaire are necessary. Second, income, social participation, working style, physical activity level, and education level were not included as correction factors. Third, although standardized health examinations were conducted, the present study included subjects who underwent health checkups at a single center in Japan and included mostly Japanese subjects. Previously, there were some reports about difference of adiponectin levels among race-ethnicity (45–47). Thus, the generalizability of results need to be verified in multi-center study. Fourth, other terms not included in our multivariable logistic regression model may also be associated with H-adiponectin/H-HOMA. Finally, this study is the epidemiological study to speculate the biological mechanism. We are not able to do the basic experiments because of clinical hospital and health-checkup center. We hope the basic experiments could be performed to clarify the molecular mechanism underlining adiponectin paradox phenomena after this epidemiological study.

## Conclusion

This study indicated that high adiponectin levels and insulin resistance may be associated with aging or low nutrition status. In addition, a mechanism of insulin resistance independent from adiponectin may be linked to low nutrition status or sarcopenia. Since renal function deteriorates withaging and insulin resistance is induced by low nutrition status or muscle loss, these factors could result in the overall effect of high adiponectin levels and insulin resistance in elderly people. Based on these findings, when we clinically evaluate adiponectin levels of elderly subjects, it is necessary to pay attention to their nutrition status and/or sarcopenia. A further longitudinal study is necessary to clarify the role of adiponectin in elderly people.

## Data Availability Statement

The datasets presented in this study can be found in online repositories. The names of the repository/repositories and accession number(s) can be found in the article/**Supplementary Material**.

## Ethics Statement

The study was approved by the human ethics committees of Sumitomo Hospital and was conducted according to the principles of the Declaration of Helsinki (approval No. 2021-38). The patients/participants provided their written informed consent to participate in this study.

## Author Contributions

JM and KK planned this study and interpreted the data. JM, TF, AM, KaS and YM recruited and collected clinical cases. JM conducted the statistical analysis of relevant data. JM and KK participated in the writing and modification of the article. YaT, KeS, YoT, YM and HR critically revised the manuscript. All authors have read and approved the manuscript.

## Conflict of Interest

The authors declare that the research was conducted in the absence of any commercial or financial relationships that could be construed as a potential conflict of interest.

## Publisher’s Note

All claims expressed in this article are solely those of the authors and do not necessarily represent those of their affiliated organizations, or those of the publisher, the editors and the reviewers. Any product that may be evaluated in this article, or claim that may be made by its manufacturer, is not guaranteed or endorsed by the publisher.
