# Supplementary material for: The Combination of High Levels of Adiponectin and Insulin Resistance Are Affected by Aging in Non-Obese Old Peoples
Source: Front Endocrinol (Lausanne). 2022 Jan 7;12:805244. doi: 10.3389/fendo.2021.805244 (PMC8777034; doi:10.3389/fendo.2021.805244)
Supplement: Supplementary file 1 [file Table_1.pdf]

SUPPLEMENT TABLE A. Logistic multivariable regression analysis for the combination of high adiponectin and high HOMA-R without kidney disease and eGFR < 60 L/min/1.73m^2.

	male (n = 2,701)		female (n = 1,784)	
	Odds ratio (95% CI)	p-value	Odds ratio (95% CI)	p-value
Age	1.04 (1.02-1.06)	<0.001	1.07 (1.05-1.10)	<0.001
Body mass Index	1.12 (1.08-1.17)	<0.001	1.08 (1.04-1.13)	<0.001
Systolic blood pressure, mmHg	1.00 (0.99-1.01)	0.213	1.02 (1.01-1.02)	<0.001
HbA1c	1.01 (0.86-1.18)	0.939	0.70 (0.52-0.93)	0.015
Triglyceride	0.99 (0.99-1.01)	0.221	0.99 (0.99-0.99)	0.048
High-density lipoprotein cholesterol	1.00 (0.99-1.01)	0.417	1.02 (1.01-1.03)	<0.001
Hemoglobin	1.05 (0.94-1.16)	0.396	1.17 (1.01-1.36)	0.034
albumin	1.07 (0.71-1.61)	0.759	0.79 (0.48-1.32)	0.371
eGFR	0.99 (0.98-1.00)	0.220	1.00 (0.99-1.02)	0.518
**Medical history**				
Coronary artery disease	1.07 (0.66-1.73)	0.780	0.45 (0.19-1.10)	0.079
Stroke	0.45 (0.20-0.99)	0.049	1.08 (0.49-2.39)	0.853

Among the final study populations (n=5,673; male 3,467, female 2,206), after excluding 1,188 individuals who had history of kidney disease and/or eGFR < 60 mL/min/1.73 m^2, subjects with eGFR ≥ 60 mL/min/1.73 m^2 and no kidney disease were total 4,485 (male 2,701, female 1,784). The median levels of adiponectin were 7.8 and 13.1 μg/mL for males and females, respectively, while median HOMA-IR values for males and females were 1.29 and 1.16. Based on these results, we divided all subjects into the H-adiponectin/H-HOMA, H-adiponectin/L-HOMA, L-adiponectin/H-HOMA, and L-adiponectin/L-HOMA groups. For males, these groups comprised 451 (16.7%), 900 (33.3%), 902 (33.4%), and 448 (16.6%) subjects, respectively, while the corresponding groups for females comprised 324 (18.2%), 570 (32.0%), 558 (31.3%), and 332 (18.6%) subjects.

* adjusted for age (y), Body mass index (kg/m2), Systolic blood pressure (mmHg), HbA1c (%), Triglyceride, High-density lipoprotein cholesterol, Hemoglobin, Albumin, estimated glomerular filtration rate (eGFR) (mL/min/1.73 m2), and medical history of coronary disease and stroke at their first visit during the study period.
