# Supplementary material for: The Combination of High Levels of Adiponectin and Insulin Resistance Are Affected by Aging in Non-Obese Old Peoples
Source: Front Endocrinol (Lausanne). 2022 Jan 7;12:805244. doi: 10.3389/fendo.2021.805244 (PMC8777034; doi:10.3389/fendo.2021.805244)
Supplement: Supplementary file 2 [file Table_2.pdf]

**SUPPLEMENT TABLE B. Logistic multivariable regression analysis for High adiponectin and High HOMA-R in 5,673 subjects (3,467 males and 2,206 females) stratified by age.**

| | 60 ≤ age <65 | | | | 65 ≤ age <70 | | | | 70 ≤ age <75 | | | | 75 ≤ age | | | |
|---|---|---|---|---|---|---|---|---|---|---|---|---|---|---|---|---|
| | Univariable | | *Multivariable | | Univariable | | *Multivariable | | Univariable | | *Multivariable | | Univariable | | *Multivariable | |
| | Odds ratio (95% CI) | *P* value | Odds ratio (95% CI) | *P* value | Odds ratio (95% CI) | *P* value | Odds ratio (95% CI) | *P* value | Odds ratio (95% CI) | *P* value | Odds ratio (95% CI) | *P* value | Odds ratio (95% CI) | *P* value | Odds ratio (95% CI) | *P* value |
| **Males** | | | | | | | | | | | | | | | | |
| age | 1.02 (0.93-1.12) | 0.681 | 1.02 (0.93-1.12) | 0.696 | 1.01 (0.89-1.14) | 0.922 | 1.00 (0.88-1.14) | 0.947 | 1.01 (0.88-1.16) | 0.860 | 1.03 (0.89-1.19) | 0.698 | 1.07 (1.00-1.14) | 0.045 | 1.07 (0.99-1.15) | 0.053 |
| BMI | 1.11 (1.05-1.16) | <0.001 | 1.14 (1.08-1.20) | <0.001 | 1.13 (1.06-1.20) | <0.001 | 1.11 (1.04-1.19) | 0.002 | 1.08 (0.99-1.17) | 0.008 | 1.08 (0.99-1.17) | 0.061 | 1.13 (1.03-1.24) | 0.009 | 1.13 (1.01-1.26) | 0.031 |
| **Females** | | | | | | | | | | | | | | | | |
| age | 1.07 (0.94-1.23) | 0.293 | 1.09 (0.96-1.25) | 0.193 | 0.93 (0.80-1.08) | 0.356 | 0.91 (0.78-1.07) | 0.252 | 0.97 (0.80-1.16) | 0.712 | 0.97 (0.80-1.17) | 0.743 | 1.06 (0.98-1.14) | 0.164 | 1.05 (0.96-1.14) | 0.277 |
| BMI | 1.07 (1.01-1.13) | 0.014 | 1.09 (1.02-1.17) | 0.007 | 1.05 (0.98-1.12) | 0.148 | 1.08 (1.00-1.17) | 0.038 | 1.03 (0.95-1.12) | 0.422 | 1.07 (0.97-1.17) | 0.174 | 1.02 (0.95-1.10) | 0.633 | 1.04 (0.96-1.13) | 0.364 |

Abbreviations: CI, confidence intercal.

* adjusted for age (y), Body mass index ($kg/m^2$), Systolic blood pressure (mmHg), HbA1c (%), Triglyceride, High-density lipoprotein cholesterol, Hemoglobin, Albumin, estimated glomerular filtration rate (eGFR) ($mL/min/1.73\ m^2$), and medical history of coronary disease and stroke at their first visit during the study period.
